# Oxidative Stress in HPV-Driven Viral Carcinogenesis: Redox Proteomics Analysis of HPV-16 Dysplastic and Neoplastic Tissues

**DOI:** 10.1371/journal.pone.0034366

**Published:** 2012-03-28

**Authors:** Federico De Marco, Elona Bucaj, Cesira Foppoli, Ada Fiorini, Carla Blarzino, Kozeta Filipi, Alessandra Giorgi, Maria Eugenia Schininà, Fabio Di Domenico, Raffaella Coccia, D. Allan Butterfield, Marzia Perluigi

**Affiliations:** 1 Laboratory of Virology. The Regina Elena Cancer Institute, Rome, Italy; 2 Department of Biochemical Sciences, Sapienza University of Rome, Rome, Italy; 3 CNR Institute of Molecular Biology and Pathology, Rome, Italy; 4 Cancer Unit - Department of Epidemiology, Institute of Public Health, Tirana, Albania; 5 Department of Chemistry, Center of Membrane Sciences, and Sanders-Brown Center on Aging, University of Kentucky, Lexington, Kentucky, United States of America; Karolinska Institutet, Sweden

## Abstract

Genital infection by high risk Human Papillomavirus (HR-HPV), although recognized as the main etio-pathogenetic factor of cervical cancer, is not *per se* sufficient to induce tumour development. Oxidative stress (OS) represents an interesting and under-explored candidate as a promoting factor in HPV-initiated carcinogenesis. To gain insight into the role of OS in cervical cancer, HPV-16 positive tissues were collected from patients with invasive squamous cervical carcinoma, from patients with High Grade dysplastic HPV lesions and from patients with no clinical evidence of HPV lesions. After virological characterization, modulation of proteins involved in the redox status regulation was investigated. ERp57 and GST were sharply elevated in dysplastic and neoplastic tissues. TrxR2 peaked in dysplastic samples while iNOS was progressively reduced in dysplastic and neoplastic samples. By redox proteomic approach, five proteins were found to have increased levels of carbonyls in dysplastic samples respect to controls namely: cytokeratin 6, actin, cornulin, retinal dehydrogenase and GAPDH. In carcinoma samples the peptidyl-prolyl cis-trans isomerase A, ERp57, serpin B3, Annexin 2 and GAPDH were found less oxidized than in dysplastic tissues. HPV16 neoplastic progression seems associated with increased oxidant environment. In dysplastic tissues the oxidative modification of DNA and proteins involved in cell morphogenesis and terminal differentiation may provide the conditions for the neoplastic progression. Conversely cancer tissues seem to attain an improved control on oxidative damage as shown by the selective reduction of carbonyl adducts on key detoxifying/pro-survival proteins.

## Introduction

Human Papillomavirus type 16 (HPV16) is the most prevalent High Risk (HR) type in premalignant and malignant cervical lesions [1]. The combined actions of its oncogenes E5, E6, E7 deregulate many cellular functions providing the conditions for genetic damage accumulation and cancer progression [2]. Experimental and epidemiological evidences however indicate that the vast majority of cervical infections regress spontaneously while only a minor part of them eventually progresses to invasive cancer. Thus HPV infection, although necessary, is not *per se* sufficient to induce cancer. Other factors have to be involved in the progression of infected cells to the full neoplastic phenotype.

The present poor knowledge about the neoplastic progression mechanisms has dramatic consequences on the clinical side. As a matter of fact based on the current screening methods, it is not possible to predict the clinical outcome of a single lesion. Thus while just a very minor part of them tend to progress all of them have to be regarded as a potentially progressive. Consequently a large number of patients have to be treated as potentially progressive patients and are therefore submitted to ultimately unnecessary surgical treatments. In the search for molecular markers able to predict the clinical outcome of dysplastic lesions many of viral, host-related and environmental factors have been taken into account and examined. Nevertheless HPV related carcinogenesis remains poorly understood and current screening protocols still wait improvements. Among environmental factors Oxidative Stress (OS), although appearing a good candidate as cancer promoting factor has been comparatively neglected so far. OS is a condition arising when the production of reactive oxygen species (ROS) is not matched by the antioxidant/repairing pathways of the cell. ROS are constantly generated in aerobic cells by the incomplete reduction of molecular O_2_ to H_2_O during mitochondrial oxidative phosphorylation, as well as during a number of processes such as inflammation, infections, mechanical and chemical stresses, exposure to UV and to ionising irradiation [3], [4]. ROS cause oxidative damage to cell membrane lipids, proteins, and nucleic acids having the potential to induce both acute and chronic degenerative processes including ageing and cancer [5], [6]. This work aims to identify new molecular markers correlating with neoplastic progression in HPV 16 transformed cervical cells. To this purpose the expression of OS related proteins and the pattern of oxidative adducts on cell proteome (the redox proteome) were assessed on cervical tissues of patients with HPV 16 infection. Overall, our results indicated that an increased oxidant environment is associated with an increased antioxidant activity in dysplastic and neoplastic tissues. Their potential role in cancer progression is discussed.

## Materials and Methods

### Patients enrolment

The study design and enrolment criteria were approved by the Regina Elena's local Ethical Committee. All participants provided full written informed consent [7]. Patients attending either the Gynecological Department of the Regina Elena Cancer Institute of Rome (Italy) or the Oncological Hospital at the University Hospital center “Mother Teresa” of Tirana (Albania) and presenting with clinically/colposcopic evidence of dysplastic lesions or invasive cervical cancer, were invited to participate to the study. Women with evidence of uterine fibroleiomyoma were also invited to participate as “non neoplastic controls”. Eligibility criteria: to be eligible patients had to be at least 18 years old, to be not pregnant, not suffering form any other disease apart the reason for asking gynecological advise, to have no/to have never suffered from other neoplastic disease. During the period, January 2008 to December 2009, 87 women were selected among those providing a full written informed consent and matching the above inclusion criteria. At entry, enrolled patients underwent a full gynecological examination. Ecto-cervical cells were collected and stored in Thin-Prep for liquid based cytology and virological assays. Histological samples were taken and divided into two halves. One used for standard histo-pathological evaluation and processed under current criteria. The second one was immediately frozen in dry ice/ethanol bath and used later for viral assay and for protein extraction. Any decision about patients' diagnosis, treatment and follow-up, was based merely on current clinical criteria irrespective of any research need.

### Protein extraction

Fresh or frozen histological samples were cut in small pieces and incubated for 20 min in ice with lysis buffer (10 mM Hepes pH 7.9, 10 mM K_2_EDTA, 5 mM NaCl, 1% TritonX-100, 10 mM â-mercapto-ethanol, aprotinine 5 mg/L). Lysates were sequentially disrupted by a mechanical mincer (Ultra-Turrax IKA T10) and by a potter device, until a fine, cloudy suspension was obtained. The suspension was then clarified in a JA-21 Beckman Super-centrifuge at 16,000 g for 20 min at +4°C. Protein concentration in the supernatant was determined by the Coomassie Plus Pierce Protein Assay (Pierce Inc., Rockford, IL, USA). Samples were then divided into aliquots and stored at −80°C until use.

### Viral analyses

DNA was extracted from a small piece of tissue by the QIAamp DNA Mini Kit (QIAGEN Gmbh, Hilden, Germany) used according to the manufacturer's instructions.

For HPV detection the samples were amplified using the MY09/MY11 primer couple [8]. Samples adequacy to PCR analysis was assessed by a parallel β-globin gene amplification with primers GH-20/PC-04 (tab. 1) as described by Saiki et al, 1988 [9]. In both cases amplification consisted in an initial step at 95°C for 150 sec for Polymerase activation, followed by 35 cycles of denaturation at 95°C for 30 sec, annealing at 56°C for 30 sec and of extension at 72°C for 40 sec plus a final cycle with a 10 min long extension for optimal chain termination. Amplified products were separated by 2.5% Etidium bromide stained agarose gel electrophoresis and visualized under UV-B trans-illumination by gel direct inspection. The HPV-16 positive Siha, CaSki and HK-168 [10] cell lines were used as positive controls. HaCaT cells were used as negative controls.

Viral typing was assessed by direct sequencing of amplified products by the BigDye Terminator 1.1 Cycle Sequencing Kit (Sanger method). Sequences were aligned to prototype viral sequence through the BLAST resource at the NCBI(http://www.ncbi.nlm.nih.gov).

The viral load of HPV-16 positive samples was determined by a SYBR Green quantitative PCR (qPCR) procedure based on the work of Roberts et al. [11] with minor modifications. Briefly total DNA was extracted and purified from 10^7^ Siha cells and adjusted to 1.0 ml final volume in Tris EDTA Buffer pH 8.0. Tenfold serial dilution were derived from the above stock solution producing a titration series of 10^4^, 10^3^, 10^2^, 10^1^, 10^0^ cell genome/µl and used to derive target-specific standard curves in every PCR session. With its 1∶1 ratio for the E6:β-globin genes this line provides an excellent reference for the simultaneous assessment in complex clinical samples of both viral copies and cellular abundance. All PCR were carried out in a IQ4 Biorad Cycler with Iq SYBR Green Supermix (both purchased from Biorad Srl, MI, Italy). Reactions were set up in 25 µl final volume containing 1× reaction mixture, 500 nM of each primer and 1 µl of standard or sample DNA. Amplification conditions consisted in: TaQ polymerase thermal activation at 95°C for 150 sec followed by 35 cycles of denaturation at 95°C for 30 sec; annealing at 56°C for 30 sec; extension at 72°C for 40 sec; sample reading at 75°C for 10 sec. A final cycle extension at 72°C for 10 min followed by a melting curve ranging from 70°C to 95°C with 0,5°C incremental temperature/10 second step were incorporated. The copy number of the viral oncogenes E6 (table 1) and the cell number (i.e: the number of human β globin copies) in experimental samples were evaluated referring the amplicon threshold cycle (assayed in duplicate) to its specific standard curve (assayed in triplicate). The primers used are listed in table 1. The average lesional Viral Load was then calculated as the E6/beta globin ratio and expressed as viral Copies per Haploid Cellular Genome (CHCG).

**Table 1:** Sequence, Tm and references of primers used.

PRIMERS	5′-3′ SEQUENCES	Bp	T_m_(C°)	reference
**MY09**	**CGTCCMARRGGAVVACTGATC**	**∼450**	**50**	Manos et al 1989
**MY11**	**GCMCAGGGVVCATAAYAATGG**		**52**	
**GH20**	**GAAGAGCCAAGGACAGGTAC**	**260**	**62**	Saiki et al 1988
**PC04**	**CAACTTCATCCACGTTCA CC**		**60**	
**16E6-S 26**	**AAGGGCGTAACCGAAATCGGT**	**207**	**64**	Nagao et al 2002
**16E6-AS 233**	**CATATACCTCACGTCGCAG**		**58**	
**16E2-S 3383**	**TTATTAGGCAGCACTTGGCCA**	**177**	**62**	Nagao et al 2002
**16E2-AS 3560**	**GTGAGGATTGGAGCACTGTCC**		**64**	
**16E2-S 2734**	**AGGACGAGGACAAGGAAAA**	**1139**	**56**	Yoshinouchi et al 1999
**16E2-AS 3846**	**GGA TGC AGT ATC AAG ATT TG**		**56**	

Names of HPV16 primers include sense of primer extension followed by the position of the 5′ nt on prototype sequence for rapid primer location.

To obtain information about the physical status of viral genome the Rolling Circle Amplification (RCA) was used [12], [13]. Such a recently implemented procedure specifically dedicated to the positive detection of episomal genomes was performed with the TempliPhi 100 Amplification Kit (Amersham Biosciences UK Limited, Amersham, UK) used according to the manufacturer's instructions.

### Western blot

Endoplasmic Reticulum protein 57 (ERp-57), glutathione S-transferase (GST), inducible nitric oxide synthase (i-NOS) and thioredoxin reductase 2 (TrxR2) levels were evaluated by Western blot analyses. Sample aliquots (40 µg of protein) were subjected to 12.5% SDS-PAGE and electroblotted (1 h at 100 V) to nitrocellulose membranes (Bio-Rad) using 25 mM Tris, 192 mM glycine and 20% (v/v) methanol. Equal protein loading was confirmed by staining with 0.2% v/v Ponceau S in 7% acetic acid. Blotted membranes were blocked with 3% albumin in T-TBS and challenged with appropriate primary antibodies, namely anti-Erp-57 rabbit polyclonal antibody, anti-GST mouse monoclonal antibody, anti-iNOS rabbit polyclonal antibody (Upstate, Millipore S.p.a, MI, IT) and TrxR2 goat polyclonal antibody (Santa Cruz Biotech. Inc., Santa Cruz, CA,USA) for 1 h at room temperature. Unbound antibodies were removed by washing twice with Tris-buffered saline containing 0.1% Tween 20, for 5 minutes. The membranes were then incubated with horseradish peroxidase-conjugated secondary antibody (Sigma–Aldrich Inc. St. Louis, MO) diluted 1∶5000. Protein bands were visualized with ECL Plus™ (Amersham) according to the manufacturer's protocol. Blots were scanned on a GS880 densitometer (Biorad) and quantified by QuantityOne image software.

### Protein oxidation measurement

Protein oxidation was measured according to Butterfield et al. [14]. Briefly, samples (5 µL) were added with 5 µL of 12% SDS and derivatized with 10 mM 2,4-dinitrophenylidrazine (DNPH) at room temperature for 20 min. Samples were neutralized with 7.5 µL of neutralization solution (2 M Tris in 30% glycerol). Derivatized samples (250 ng) were then blotted onto a nitrocellulose membrane under vacuum using a slot-blot apparatus (Bio-Rad). Membranes were blocked with 3% BSA in TBS-T for 1 h and next incubated with rabbit antibody to protein-bound DNP (diluted 1∶150) for 90 min. After washing with TBS-T, membranes were incubated with anti-rabbit IgG alkaline phosphatase secondary antibody (1∶5000) in TBS-T for 1 h at room temperature. The membrane was washed in TBS-T and developed using a solution of NBT (0.2 mM) and BCIP (0.4 mM) in alkaline phosphate buffer (0.1 M Tris, 0.1 M NaCl, 5 mM MgCl_2_; pH 9.5). Dried blots were quantified using QuantityOne image analysis (Bio-Rad).

### 2D electrophoresis and western blot

For the first-dimension electrophoresis, proteins (200 µg in 200 µL of rehydration buffer) were applied to a ReadyStrip™ IPG strip pH 3–10 (Bio-Rad). The strips were soaked in the sample solution for 1 h to allow uptake of the proteins. The strips were then actively rehydrated in Protean IEF Cell Apparatus (Bio-Rad) for 16 h at 50 V. The isoelectric focusing was performed at 300 V for 2 h linearly; 500 V for 2 h linearly; 1000 V for 2 h linearly, 8000 V for 8 h linearly and 8000 V for 10 h rapidly. All the processes above were carried out at room temperature. The focused IEF strips were stored at −80°C until second dimension electrophoresis was performed.

For second dimension electrophoresis, thawed strips were equilibrated for 10 min in 50 mM Tris-HCl (pH 6.8) containing 6 M urea, 1% (w/v) sodium dodecyl sulfate (SDS), 30% (v/v) glycerol, and 0.5% dithiothreitol, and then re-equilibrated for 15 min in the same buffer containing 4.5% iodacetamide in place of dithiothreitol. 12% Precast criterion gels (Bio-Rad) were used to perform second dimension electrophoresis. Precision Protein™ Standards (Bio-Rad) were run along with the sample at 200 V for 65 min. After electrophoresis, the gels were fixed (7% acetic acid, 10% methanol) and stained with Bio-Safe Coomassie Gel Stain (Bio-Rad).

To identify carbonylated proteins, samples (200 µg proteins) were derivatized as above described, subjected to 2-DE and transferred to nitrocellulose membrane using Criterion Blotter apparatus (Bio-Rad) at 100 V for 1 h. The carbonylated proteins were detected as above reported.

### Image Analysis

The 20 gels (n = 7 controls, n = 6 dysplasia and n = 7 carcinoma) and 20 nitrocellulose blots were scanned and saved in TIF format using a GS-800 densitometer (Bio-Rad). PDQuest 2D Analysis software (version 7.2.0, Bio-Rad) was used for matching and analysis of visualized protein spots among differential gels and membranes. The anti-DNP immune-reactivity of individual proteins was normalized to protein content evaluated by the intensity of Coomassie blue stained spots. After completion of spot matching, the normalized intensity of each protein spot from individual gels was compared among the groups using statistical analysis. Statistical significance was assessed by a two-tailed Student's *t*-test, the method of statistical analysis most appropriate for proteomic analysis of small number of protein spots [15]. P values <0.05 were considered significant for comparison between control and experimental data.

### Trypsin digestion and protein identification by mass spectrometry

Selected spots were manually excised from gel and submitted to trypsin proteolysis [16]. MALDI-ToF MS analyses were performed in a Voyager-DE STR instrument (Applied Biosystems, Framingham, MA, USA) equipped with a 337 nm nitrogen laser and operating in reflector mode. Mass data were obtained by accumulating several spectra from laser shots with an accelerating voltage of 20 kV. Two tryptic autolytic peptides were used for the internal calibration (*m/z* 842.5100 and 2807.3145). Data were analysed by MoverZ program (v. 2002, http://bioinformatics.genomicsolutions.com), according to default parameters. Identification by peptide mass fingerprint (PMF), with the mono-isotopic mass list, after exclusion of expected contaminant mass values by Peak Erazor program (http://www.protein.sdu.dk/gpmaw/Help/PeakErazor/peakerazor.html), was performed using the Mascot search engine (v. 2.3) against human SwissProt database [(SwissProt 2011_08 (531473 sequences; 188463640 residues)]. Up to one missed cleavage, 50 ppm measurement tolerance, oxidation at methionine (variable modification) and carbamidomethylation at cysteine (fixed modification) were considered. Identifications were validated when the probability-based Mowse protein score was significant according to Mascot [17].

### GAPDH activity assay

GAPDH activity was measured by a colorimetric assay kit (ScienCell, Research Laboratories Co, Carlsbad, CA). The method is based on the oxidization of â-NADH to â-NAD in the presence of 3-phosphoglyceric acid (3-PGA), adenosine 5′-triphosphate (ATP) and GAPDH. The GAPDH activity is determined by assaying the rate of NADH oxidation, which is proportional to the reduction in absorbance at 340 nm over time (A_340 nm/min_). Briefly, 5 µl of each sample or standard is added to each well, in the 96-well plate, containing 145 µl of GAPDH assay mixture, and the A_340 nm_ kinetic was measured. Enzyme activity is calculated as U.A./mg protein.

#### Determination of 8-hydroxy-2-deoxy Guanosine (8-OH-dG)

DNA oxidation was evaluated by the 8-OH-2deoxy Guanosine EIA kit (StressMarq Biosciences Inc, Victoria BC CANADA) used according to the manufacturer's instructions.

### Chemicals and biochemical

All other materials used unless otherwise specified were analytical grade products purchased from the current laboratory suppliers either Sigma–Aldrich (St. Louis, MO, USA) or Bio-Rad (Bio-Rad Laboratories, Milan, Italy).

### Statistical analysis

Two-sided, Student's *t*-tests were used to analyze differences in protein levels between dysplasia and carcinoma. A *p*-value of less than 0.05 was considered statistically significant. The significance of the change in carbonylation of specific proteins in the proteomics study was evaluated via nonparametric Mann-Whitney-Wilcoxon test. *P* <0.05 was considered statistically significant.

## Results

### Patients

During the period from January 2008 to December 2009 a total of 87 patients yielded their consent to participate to the study. Among them 35 had an invasive squamous cell carcinoma (SCC), 1 an adeno-carcinoma, 12 were affected by a cervical dysplastic lesion and 23 were suffering for a uterine fibroleiomyoma. The remaining 15 patients turned out to be affected by other inflammatory or chronic/degenerative pelvic diseases and were excluded from further analyses. Viral typing showed that HPV16 was present in 25/35 patients with invasive SCC, in 6/12 patients with dysplastic lesion and in 7/23 patients with uterine fibroleiomyoma. These latter, for the sole purpose of this work are here considered as control patients. All the HPV16 patients, listed in table 2 entered the study and were further characterized. The major clinical features concerning tumour staging and grading are also reported together with viral determinats.

**Table 2:** List of HPV-16 positive patients together with their major clinical, virological and pathological determinants.

ID	Age	DNAviral load	RCA	Cytology	Kolposcopy	Histology	Stage (FIGO)	Hist Grade	LymphNode Met	DistantMet
Z A	51	3,17×10^−3^	ND	Normal	ND	Negative	-	-	-	-
E L	44	3,00×10^−4^	ND	Normal	ND	Negative	-	–	–	-
V M	32	2,19×10^−2^	ND	Normal	ND	Negative	-	-	-	-
Q M	50	3,50×10^−2^	Positive	Normal	ND	Negative	-	-	-	-
H C	55	1,98×10^−3^	ND	Normal	ND	Negative	-	-	-	-
H S	51	2,30×10^−3^	ND	Normal	ND	Negative	-	-	-	-
B Z	51	3,11×10^−3^	ND	Normal	ND	Negative	-	-	-	-
D H	48	1,52×10^−2^	ND	HSIL	ANTZ G1	CIN-II	-	-	-	-
E M	35	7,60×10^−3^	ND	HSIL	ANTZ G2	CIN-III	-	-	-	-
M B	40	3,30×10^−3^	ND	HSIL	ANTZ G2	CIN-III	-	-	-	-
M T	31	9,2×10^−3^	ND	HSIL	ANTZ G2	CIN-II	-	-	-	-
L B	43	6,68×10^−2^	ND	HSIL	ANTZ G1	CIN-III	-	-	-	-
T S	39	4,27×10^−3^	ND	HSIL	ANTZ G2	CIN-III	-	-	-	-
M R	55	1,1×10^2^	Positive	HSIL/worse	ANTZ G2	Invasive SCC	IIA	G3	No	No
D A	48	5,60×10^1^	Positive	HSIL/worse	ANTZ G2	Invasive SCC	IIB	G3	No	No
M S	64	2,00×10^1^	ND	HSIL/worse	ANTZ G1	Invasive SCC	IB1	G2	No	No
C G	44	1,81×10^1^	ND	HSIL/worse	ANTZ G2	Invasive SCC	IIB	G3	No	No
D′A A	68	9,20×10^−4^	ND	HSIL/worse	ANTZ G1	Invasive SCC, with basaloid aspects	IIA	G2	No	No
G I	40	5,88×10^0^	ND	HSIL/worse	ANTZ G2	Invasive EC	IIB	G3	No	No
N B	59	3,00×10^2^	ND	HSIL/worse	ANTZ G2	Invasive SCC,	IIB	G3	No	No
F R	52	6,23×10^−1^	ND	HSIL/worse	ANTZ G2	Invasive EC	IIB	G3	Yes	No
S C	47	2,35×10^2^	ND	HSIL/worse	ANTZ G2	Invasive SCC,	IIB	G3	No	No
A M	43	1,22×10^1^	ND	HSIL/worse	ANTZ G2	Invasive SCC,	IA1	G3	No	No
P B	45	3,00×10^0^	ND	HSIL/worse	ANTZ G2	Invasive SCC,	IIA	G2	No	No
C C	46	4,60×10^2^	ND	HSIL/worse	ANTZ G2	Invasive SCC,	IIA	G3	No	No
A M	52	6,50×10^2^	ND	HSIL/worse	ANTZ G2	Invasive SCC,	IIB	G2	No	No
P MC	55	5,4×10^1^	ND	HSIL/worse	ANTZ G2	Invasive SCC,	IIA	G3	No	No
G.R	73	3,60×10^−3^	ND	HSIL/worse	ANTZ G2	Invasive EC	IIA	G2	Yes	No
A.LE	70	7,80×10^0^	ND	HSIL/worse	ANTZ G2	Invasive SCC,	IB2	G3	No	No
M P	58	1,25×10^3^	ND	HSIL/worse	ANTZ G2	Invasive SCC	IB2	G2	No	No
M M	75	7,30×10^−2^	ND	HSIL/worse	ANTZ G1	Invasive SCC,	IB2	G2	No	No
P E	53	1,7×10^0^	ND	HSIL/worse	ANTZ G2	Invasive SCC,	IIB	G3	No	No
M M	56	7,43×10^2^	ND	HSIL/worse	ANTZ G2	Invasive EC	IB2	G2	No	No
V A	49	4,91×10^−2^	ND	HSIL/worse	ANTZ G2	Invasive SCC,	IIA	G3	Yes	No
M GM	51	6,90×10^1^	ND	HSIL/worse	ANTZ G2	Invasive SCC,	IIB	G2	Yes	No
D′A L	47	1,3×10^−1^	ND	HSIL/worse	ANTZ G2	Invasive SCC,	IIA	G3	No	No
T E	59	8,3×10^2^	ND	HSIL/worse	ANTZ G2	Invasive SCC,	IA2	G3	No	No
M R	56	7,70×10^0^	ND	HSIL/worse	ANTZ G2	Invasive SCC,	IB1	G2	No	No
HK-168	-	2.8×10^0^	ND	ND	-	HPV-16 *in vitro* transformed human keratinocytes	-	-	-	-
SiHa	-	1.09×10^0^	ND	ND	-	Invasive SCC cell line	-	-	-	-
Caski	-	655×10^2^	ND	ND	-	Invasive SCC cell line	-	-	-	-
HaCaT	-	ND	ND	ND	-	Spontaneously Immortalized human keratinocytes cell line	-	-	-	-

ID: patient's identification code; DNA viral load was calculated as the E6/beta globin ratio and expressed in copy per haploid cellular genome CHCG; HSIL: High grade Squamous Intraepithelial Lesion; ANTZ: Abnormal Transformation Zone; Histological Grade: G1 Well differentiated; G2 Moderately differentiated; G3 Poorly differentiated. Met: metastases. ND: none detected; SCC samples selected for redox-proteomics analysis are boxed.

### Viral load and viral genome physical status

**Figure 1:**
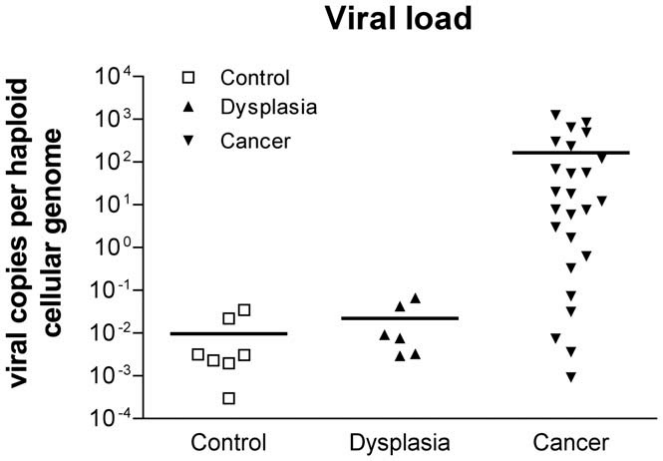
Viral load. Viral loads in CTR, DYS and SCC cervical tissues are expressed as E6 copies/β-globin copies/cell.

Viral load and viral genome physical status have been claimed to be relevant determinants in HPV infection outcome and in clinical evaluation of dysplastic and neoplastic lesions. Various methodologies are described for the evaluation of viral load. We worked with the SYBR Green method following the recommendation of Roberts et al. [11] with modifications. The E6 and the humanbeta globin gene quantification was based on a standard curve generated by a logarithmic dilution series of Siha cells, a cell line known to host a single, integrated, almost complete HPV16 CHCG [11], [18]. Primers with close annealing temperature and generating amplicons of similar length were chosen to keep to a minimum the bias due to differential target amplification (table 1). The HPV-16 E6 primers encompassed the region from nucleotide (nt) 26 to nt 233 generating a 207 bp long amplicon [19]. The primers for beta-globin (GH-20 and PC04) spanned the start codon of the human beta-globin amplifying a 268 bp tract. The viral load obtained for each specimen are listed in table 2 and plotted in figure 1. As it can be seen, in control tissue (i.e.: cervical tissue devoid of clinically evident dysplastic lesion) a mean viral load of 0.96×10^−2^ CHCG was found. All but one single value clustered close to the mean value and all of them were clearly below the 10^−1^ CHCG level. A very similar finding was obtained for dysplastic samples with a mean viral load of 2.20×10^−2^ CHCG and much less scattered values. Conversely a sharply higher mean value of 1.65×10^2^ viral CHCG was observed among invasive cancer although values were widely dispersed across seven orders of magnitude (from 10^−4^ up to 10^3^ CHCG). The HK-168, Siha and CaSki cell lines, here used as low and high ratio positive control consistently yielded the expected values of around 10^0^; 10^0^ and 10^2^–10^3^ viral CHCG respectively.

The physical status of viral genomes in the samples is reported in table 2. As it can be seen, with the RCA method, a method specifically dedicated to the direct detection of circular DNAs, episomal forms were positively identified just in one normal sample and in two neoplastic ones.

### Expression levels of stress response proteins

**Figure 2:**
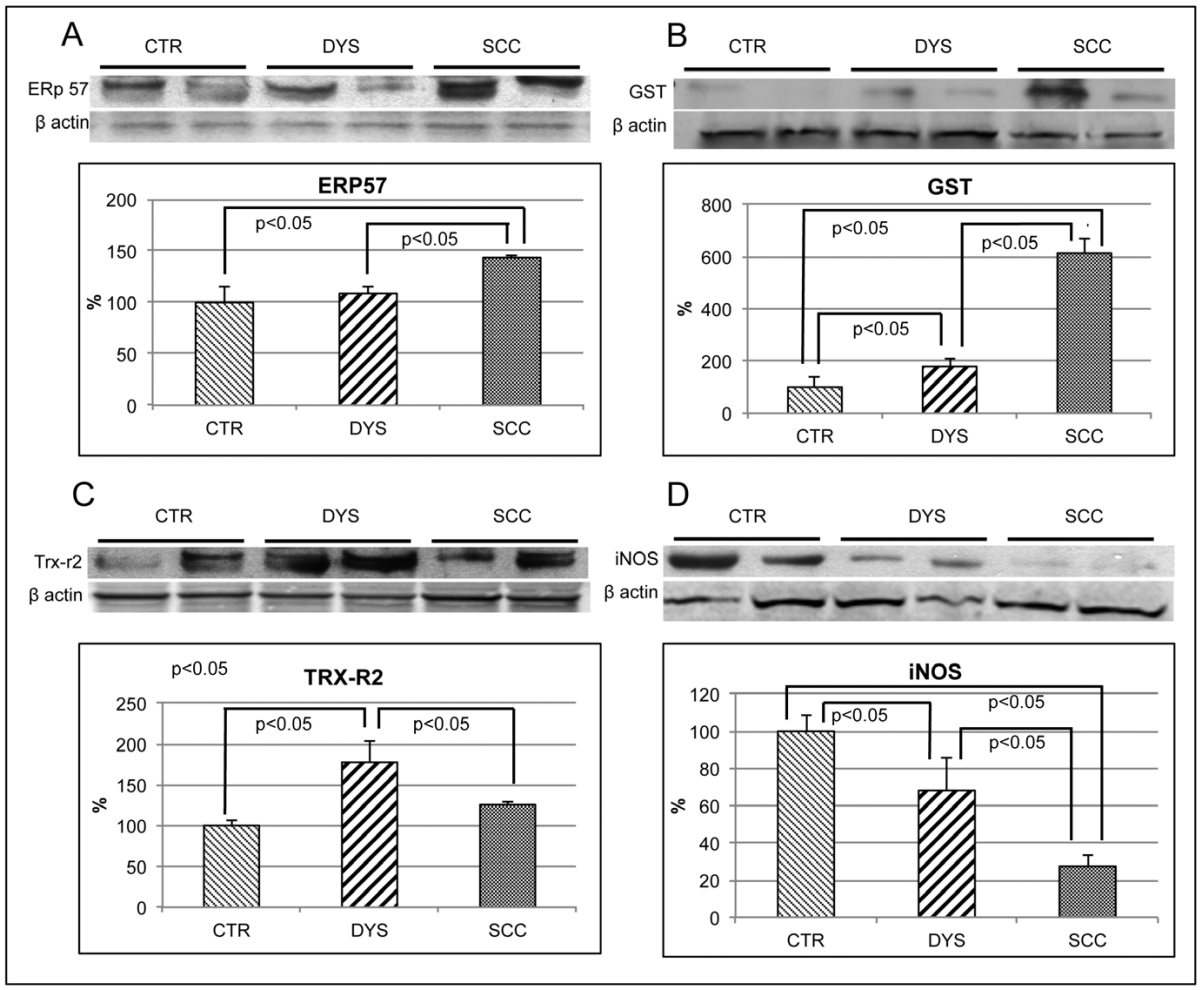
Expression levels of stress markers (ERp57, GST, TRX-R2 and iNOS). Protein expression levels in CTR, DYS and SCC cervical tissues were measured by Western blot analysis using specific antibodies for ERp57 (A), GST (B), TRX-R2 (C) and iNOS (D). Immunoblots were scanned by densitometry and all values were normalized to β-actin levels. Densitometric values shown are given as percentage of the control group, set as 100%. Data are expressed as mean ± SEM. *p*<0.05 versus control (Student's *t*-test).

Expression levels of selected stress response proteins, including Endoplasmic Reticulum protein 57 (ERp57), Glutathione S-Transferase (GST), inducible Nitric Oxide Synthase (iNOS) and mitochondrial Thioredoxin Reductase (TrxR2) were evaluated in control, dysplastic and neoplastic tissues (figure 2).

Erp57 is an ER stress marker and our previous work showed that it is a selective target of OS in epithelial cells [20]. In neoplastic tissues the expression level of ERp57 was significantly increased compared with both dysplastic and control tissues (figure 2, panel A).

GST is a detoxifying enzymes found to be overexpressed in different tumors, though no data are available in cervical cancer. Further, growing studies proposed that GST polymorphism is a candidate risk factor for developing cervical cancer. GST was sharply increased in dysplastic and in neoplastic cells compared with controls (up to 1.8 and 6 fold respectively) (figure 2, panel B).

The TrxR2, participates in mitochondrial redox signaling events and it has been recently regarded as a cancer development [21].The TrxR2, as compared with control tissue, was significantly increased in dysplastic lesions and, to a lower extent, in neoplastic lesions too (175% and 125% respectively).

i-NOS, the inducible isoform of Nitric Oxide Synthase, is a well-established marker of nitrosative stress and inflammation. Its specific role in tumor biology is still under debate. iNOS was found to be progressively decreased in dysplastic (65% of control) or neoplastic (25% of control) samples as compared with control tissues (figure 2, panel D).

### Protein oxidation

**Figure 3:**
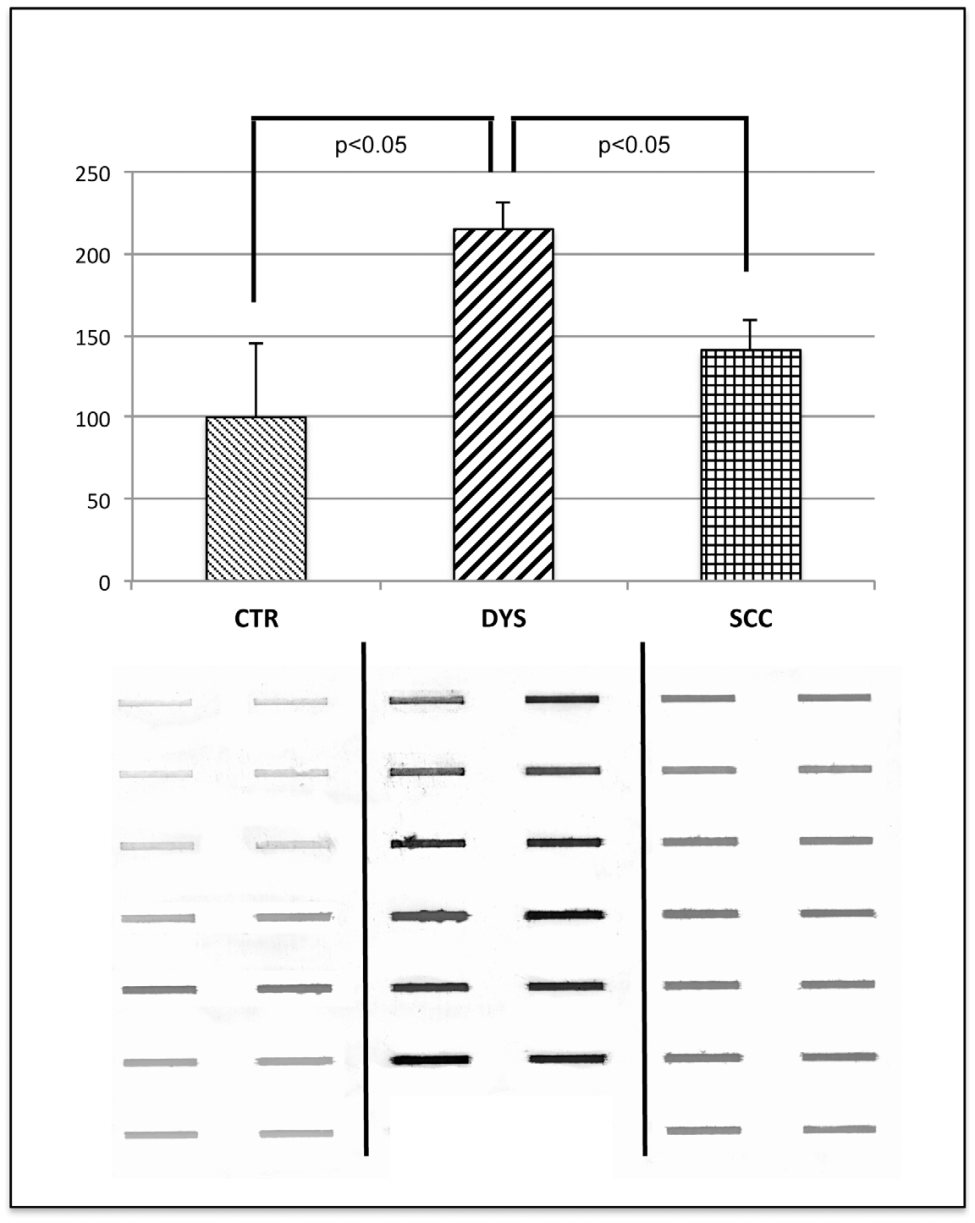
Total protein oxidation. Top: Quantification of levels of protein carbonyls in CTR, DYS and SCC cervical tissues. Samples were probed with anti-DNP protein adducts polyclonal antibody as described in *Material and Methods*. Densitometric values shown are given as percentage of the control group, set as 100%. Data are expressed as mean ± SEM. *p*<0.05 versus control (Student's *t*-test). Bottom: Protein carbonyl slot- blots from CTR, DYS and SCC samples.

To assess the extent of total protein oxidation, protein carbonyl levels were evaluated by slot-blot analysis (figure 3). Protein carbonyls were significantly increased in dysplastic tissues respect to control samples, while the levels detected in neoplastic tissues were surprisingly similar to control ones.

### Identification of carbonylated proteins

For the redox proteomics analysis, a set of 7 SCC samples were selected from those reported in table 2, to be compared, with the 6 dysplastic tissues and the 7 control ones.

**Figure 4:**
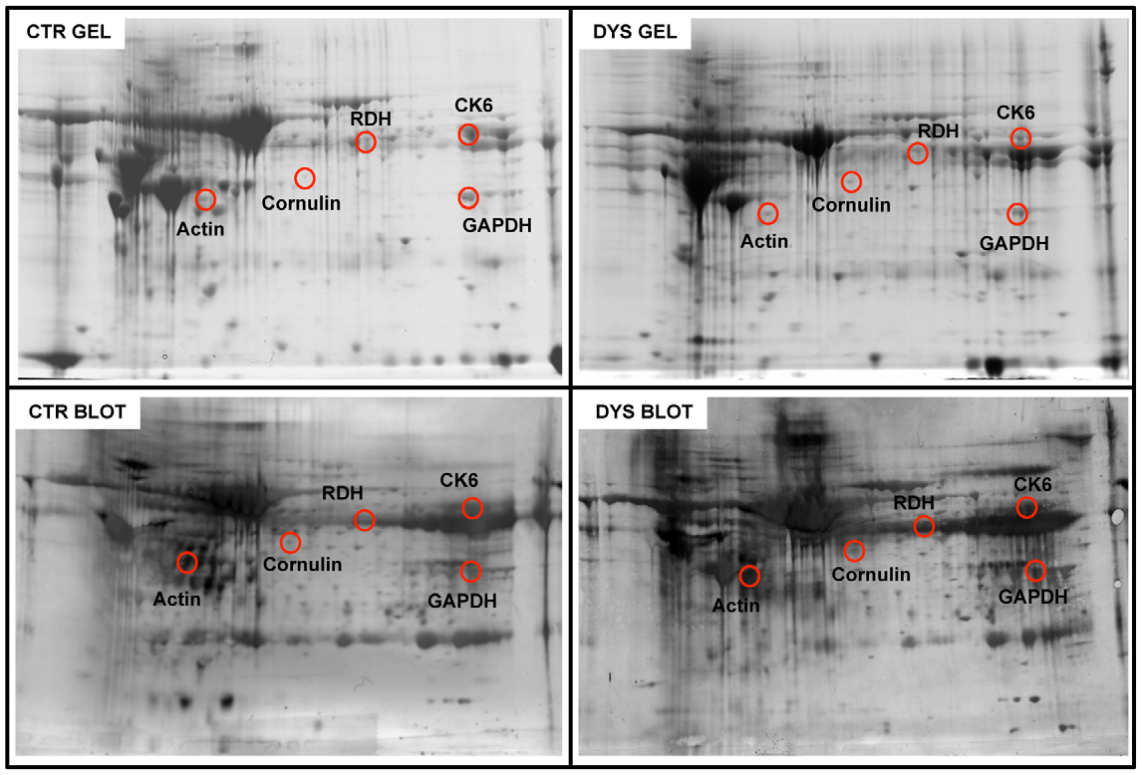
Oxidized protein detection by redox proteomics (DYS vs. CTR). Top: 2D maps of CTR (left) and DYS (right) cervical tissues. Proteins (300 µg) were separated in first dimension (pH 3–10 linear IPG); second dimension was performed on slab gel (12% gradient SDS-PAGE). Protein detection was achieved using Biosafe Coomassie staining. Bottom: 2D carbonyl immunoblots of CTR (left) and DYS (right) cervical tissues. The spots showing significant increased carbonyl levels are labeled. Relative change in carbonyl immune-reactivity, after normalization of the immunostaining intensities to the protein content, was significant for five spots. The identified proteins are listed in table III.

**Figure 5:**
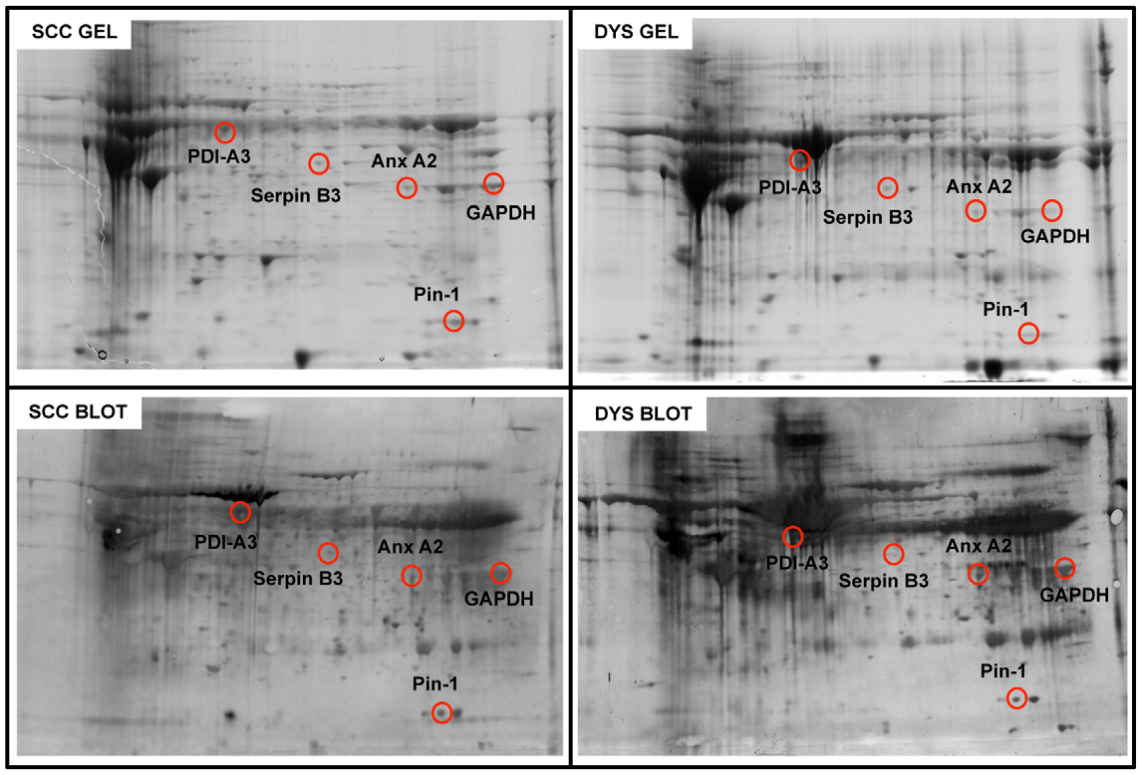
Oxidized protein detection by redox proteomics (DYS vs. SCC). Top: 2D gel maps of DYS (right) and SCC (left) cervical tissues. Protein (300 µg) were separated in first dimension (pH 3–10 linear IPG); second dimension was performed on slab gel (12% gradient SDS-PAGE). Protein detection was achieved using Biosafe Coomassie staining. Bottom: 2D carbonyl immunoblots of DYS (right) and SCC (left) cervical tissues. The spots showing significant increased carbonyl levels are labeled. Relative change in carbonyl immune-reactivity, after normalization of the immunostaining intensities to the protein content, was significant for five spots. The identified proteins are listed in table III.

Two representative 2D gels, and the corresponding blots, from control and dysplastic samples are pictured in figure 4 and 5. MS identification of proteins, along with the peptide hits, sequence coverage, Mw and pI values and the increase of specific carbonyl levels, indexed as fold oxidation compared with controls, are reported in table 3. 5 proteins were found to be more oxidized in dysplastic tissues compared with controls, namely in cytoskeletal Keratin 6 (CK 6, fragments/isoforms A,B & C), Cornulin, Actin, GAPDH and Retinal Dehydrogenase (RDH) (figure 4). These are proteins involved in cytoskeleton scaffolding (keratins and actins) and epidermal differentiation (cornulin, RDH).

**Table 3:** List of oxidized proteins successfully identified by mass spectrometry along with the peptide hits, sequence coverage, Mw and pI values and the increase/decrease of specific carbonyl levels, indexed as fold oxidation.

Protein	Swiss Proteincode	TheoricalMw/pI	SequenceCoverage %	Score	Foldoxidation
**DYSPLASIA VS. CONTROLS**					
Keratin, type II cytoskeletal 6AKeratin, type II cytoskeletal 6BKeratin, type II cytoskeletal 6C	P02538P48668P04259	60293/8.0960273/8.0960315/8.09	535049	327283311	+9.08
Glyceraldehyde-3-phosphate dehydrogenase	P04406	36201/8.57	46	131	+8.62
Cornulin	Q9UBG3	53730/5.73	48	229	+4.26
Retinal Dehydrogenase 1	P00352	55454/6.30	27	125	+31.68
Actin, cytoplasmic 1Actin, cytoplasmic 2	Q96HG5P63261	42052/5.2942108/5.31	7979	2727	+9.06
**SCC VS. DYSPLASIA**					
Glyceraldehyde-3-phosphate dehydrogenase	P04406	36201/8.57	41	112	−10.57
Peptidyl-prolyl cis-trans isomerase A	P62937	18229/7.68	41	79	−313.34
Erp 57	P30101	57146/5.98	22	96	−16.55
Serpin B3	Q8IXI3	44594/6.35	187	37	−5.18
Annexin A2	P07355	38808/7.57	45	168	−106.75

Two proteins were identified in the same spot: the fragments/isoforms A,B & C of cytoskeletal Keratin 6 (with a score of 327, 283 and 311 respectively) and the pyruvate kinase with a score of 66 (around the threshold limit).

Redox proteomics analysis was also performed to compare dysplastic and neoplastic samples. Results indicated that five proteins, namely Serpin B3, Annexin 2 (Anx2), ERp57 and peptidyl-prolyl cis/trans isomerase (Pin1) and GAPDH were less oxidized in neoplastic samples compared with dysplastic ones (figure 5). Surprisingly, oxidized proteins did not show any significant elevation in SCC tissues compared with controls.

Table 3 shows the proteins successfully identified by mass spectrometry along with the peptide hits, sequence coverage, Mw and pI values and the increase/decrease of specific carbonyl levels, indexed as fold oxidation.

#### GAPDH activity

In order to see the effect of oxidative modification on protein function, the enzymatic activity of GAPDH has been measured in dysplastic and neoplastic tissues compared with healthy controls. We found that dysplastic tissue had a lower enzymatic activity compared with controls (normalized to protein expression levels) while a recovery of the activity was evidenced in SCC samples (Table 4). These data result from the combined modification of both protein expression levels and protein oxidation and confirms that increased GAPDH carbonylation leads to impaired protein function.

**Table 4:** Expression levels, carbonyl levels and enzyme activity of GAPDH in controls (CTR), dysplastic (DYS) and carcinoma tissues (SCC) are reported.

	CTR	DYS	SCC
**GAPDH** **Expression levels**(fold vs. CTR)	1±0.1	1.6±0.3	2.4±0.2
**GAPDH** **Carbonylation levels**(fold vs. CTR)	1±0.3	9.0±0.4	1.2±0.3
**GAPDH** **Enzymatic activity**(uA/mg)	45±5.4(100±12%)	26.8±6.3(59.5±14%)	52.8±4.9(117.3±11%)

Levels are expressed as fold increase/decrease vs CTR. Enzyme activity is calculated as A_340_/mg protein and also as % vs CTR. Data are expressed as mean ± SEM.

#### 8-OH-dG

In order to support data on protein oxidation with results obtained from alternative approaches the extent of DNA oxidation was assessed by the levels of 8-OH-dG. This is an established marker of DNA oxidative damage in response to increased OS and ROS production. As it can be seen in Table 5, dysplastic tissue showed elevated level of 8-OH-dG whereas neoplastic samples displayed a surprising diminished levels as compared with both controls and dysplastic tissues.

**Table 5:** 8-OH-dG levels in control (CTR), dysplastic (DYS) and carcinoma tissues (SCC) are reported as ng of 8-OH-dG/mg DNA.

8-OH-dG	CTR	DYS	SCC
**ng/mg DNA**	168±14	213±18	60±8

Data are expressed as mean ± SEM.

## Discussion

Aiming to identify new candidate markers able to predict the clinical outcome of lesions it was necessary to compare lesions with highly homogeneous clinical and biological features. Thus considering that viral load and viral genome physical status have been assumed to be most relevant determinants in cervical infection outcome [18], [22], [23], samples were carefully evaluated under these two aspects. As above reported in normal samples, as well as in dysplastic samples, a mean viral load of roughly 10^−2^ CHCG was found while the sharply higher viral load of 1,65×10^2^ CHCG was revealed in SCC samples. These data are in agreement with the cut off value recently proposed by Carcopino *et al.* [24] for HPV-16 progressive lesions and indicate that the groups of lesions were highly homogeneous and well distinct from each other. Moreover, the physical status of viral genome is consistent with data reported by other authors showing that viral integration is not a stringent requisite for neoplastic growth, which frequently occurs in cells hosting episomal viral genomes [25].

Remarkable differences were shown in stress response markers among the three groups of lesions. The ERp57 was found to be significantly up-regulated only in neoplastic tissue compared with both dysplastic and controls tissues. The ERp57 is a ER resident member of the protein-disulphide isomerase family, which assists the maturation and transport of unfolded secretory proteins by facilitating disulphide bond formation and rearrangement reactions. ERp57 expression is induced during neoplastic transformation [26] possibly leading to redox-dependent modulation of cancer relevant regulatory factors [27], [28]. GST expression was found to be induced in dysplastic tissues and even more in neoplastic tissues. Considering that various classes of GST iso-enzymes are overexpressed in human tumour cell lines of different histological origin [29] it is likely that such an elevation represents an adaptive response mechanism, devoted to the detoxification of oxidative stress related harmful metabolites.

The TrxR2 is part of an important mechanism for maintaining the reduced intracellular environment. In addition to its possible implication in many aspects of cancer biology [30] its specific role in protein oxidative damage repair indicate that TrxR2 induction represents an adaptive response against a condition of increased ROS generation as it may occur during cancer growth [31].

The inducible form of NOS has been commonly associated with malignant diseases, however its role in carcinogenesis and tumor biology is far from being clarified. Our results indicated that compared with the level found in control tissues, iNOS expression was gradually reduced in dysplastic and neoplastic lesions. Though divergent from what observed in most cancer types, this finding is an agreement with the results by Mazibrada *et al.* [32] reporting that iNOS expression was significantly reduced from low to high-grade cervical lesions.

Taken together the above data support the view that highly active detoxifying systems (ERp57; TrxR2; GST) and reduced iNOS might be part of a complex adaptive metabolic profile allowing cell survival in an increasingly oxidant environment.

In order to better understand the role of OS in cervical cancer, we measured the extent of total protein oxidation. We found that protein carbonyls were significantly increased in dysplastic tissues, while levels detected in neoplastic tissues were not significantly different to control ones. This unexpected trend was also paralleled by the extent of oxidative DNA damage. Indeed we found that 8-OH–dG levels were clearly increased respect to both SCC and controls. These findings support the view that dysplastic state is highly vulnerable to oxidative damage, a major factor of genetic instability, providing the conditions for the neoplastic evolution of transformed cells. Conversely established tumours seem to fit well with stress conditions.

Since it has been demonstrated that protein oxidation results in diminished, complete loss of, or a toxic gain in protein function [33], the identification of specific proteins, which are irreversibly modified by carbonylation, could shed light on the molecular mechanism targeted by OS in cell transformation and tumor development. By redox proteomics analysis, in dysplastic tissue compared with controls five proteins were identified with increased carbonylation levels, namely CK6, actin, cornulin, RDH and GAPDH.

Cytokeratins (CKs) contribute to cytoskeleton organization and are well known markers of cell differentiation both in normal and neoplastic epithelia [34]. Their interaction with HPV transforming activity has been reported [35] and their quantitative detection has been proposed as an adjunct to current histological evaluation [36]. Recently, Arnouk et al. reported a decreased expression of CK6A and other CKs in HSIL and cervical cancer [37]. Our results indicate that in addition to the altered pattern of expression of CKs, a further level of cytoskeleton derangement seems to occur in dysplastic lesion in consequence of the oxidative alteration of CK6. Such a deregulation is potentially reinforced by the parallel increased oxidation of actin which cooperates with CKs in structural and functional cell shaping [38]–[39].

Cornulin is a calcium binding protein member of the fused-gene family [40] normally expressed in late epidermal differentiation and currently used as a marker of differentiation [41]. Recently cornulin has been reported to be severely reduced in cervical dysplastic lesions and to be almost completely suppressed in neoplastic lesions [37]. Our results show that in dysplastic lesion its residual amount is likely to be further impaired due to its increased level of oxidation, This result is consistent with the hypothesis, proposed by Arnouk *et al.* [37] that cornulin is a promising candidate marker that correlates with the neoplastic progression.

The RDH is an enzyme involved in the synthesis of retinoic acid, a fundamental regulator of cell differentiation, embryogenesis, tissue homeostasis and renewal [42]. Several studies reported retinoid implications in HPV related neoplastic diseases including suppressive control on integrated E6 & E7 oncogenes [43], [44]. The increased oxidation of RDH suggests its altered/reduced activity in dysplastic lesion with possible decreased control on cell differentiation maintenance as well as on pro-survival and anti-apoptotic activity of integrated viral oncogenes.

Taken together the above data indicate that in dysplastic lesions a selective oxidation of CK6, Actin, Cornulin and RDH, concur to impair their functions contributing to cytoskeleton derangement, suppression of terminal differentiation and reduced control on viral oncogenes activity.

Comparative analysis between SCC and CTR tissues did not reveal any significant increase of carbonylated protein. This result is consistent with total protein oxidation levels. However, further larger studies are needed to clarify such an apparent enigma.

Comparing the redox proteomics pattern of dysplastic and neoplastic tissues a number of proteins exhibited a lower oxidation in neoplastic tissues namely the ERp57; Anx2; Serpin B3; Pin1 and GAPDH.

Interestingly, the increased expression of ERp57 in neoplastic tissues is also associated with its reduced oxidation possibly resulting in a much greater increase of activity than expected based on purely quantitative data. This result underscores the relevance that elevated protein folding/unfolding activity may have for cell survival of cancer cells and suggests that the achievement of the neoplastic phenotype is accompanied by the activation of compensatory mechanisms able to counteract oxidative damage of selected targets and allowing the cell to fit to the hostile environment.

A similar lower level of carbonylation in tumor tissues compared with dysplastic lesions was also showed for Anx2. Recent studies suggest that Anx2 might be linked to carcinogenesis through its implication in the invasion and neovascularisation processes [45] and that the protein is regulated by the cellular redox status [46]. We have previously reported that Anx2 is oxidized in NHEK and in HPV-transformed keratinocytes upon exposure to oxidative stress [16], [20]. However, tumour cells once adapted to a more oxidized environment have improved antioxidant/detoxifying mechanisms and therefore are able to survive under stress. Thus, neoplastic cells are paradoxically able to protect themselves from oxidative damage.

Serpin B3, also known as Squamous Cell Carcinoma Antigen 1, is a serine protease inhibitor involved in regulation of plasminogen activation, inhibition of inflammation and promotion of epithelial proliferation [47]. It binds directly to the HPV16-E7 antigen and interestingly is overexpressed in tumour cells and correlate with tumour progression and it is believed to act as a molecular switch that inhibits cell death [48]. Our results suggest that reduced oxidation of serpin B3 in tumours might favor tumour cell proliferation.

Pin1 [named after the acronym: Protein Interacting with NIMA ( = Never In A Mithosis)] is indeed a Peptidyl prolyl cis-trans isomerase that isomerizes phospho-Serine/Threonine-Proline [p(S/T)-P] motifs causing them to twist between two completely distinct conformations. Pin1 is required for cell division, regulates the cell cycle and, once over-expressed, can promote oncogenesis through a number of signaling pathways [49]. The finding that Pin1 is less oxidized in tumor tissue compared with dysplasia may further correlate with the ability of tumor cells to withstand high levels of ROS by protecting several cellular component from oxidative insult.

GAPDH has long been considered a “simple” glycolitic enzyme and has been widely (and erroneously) used as an internal standard reference for RNA expression. Indeed GAPDH is tightly regulated at both transcriptional and post-translational level [50], [51]. In addition to its conventional metabolic role many other functions have been identified so far implying the participation of GAPDH to many cell function including cell adhesion and motility, endocytosis and nuclear membrane assembly, ER and Golgi trafficking, tRNA export, DNA repair, telomere protection and cell death [52], [53]. We found increased oxidation of GAPDH in dysplastic lesions vs control tissues while decreased oxidation was evident in neoplastic tissues vs dysplasia. The increased oxidation/dysfunction in dysplasia leads to decreased enzyme activity. Conversely, in neoplastic cells a reduced levels of oxidation was coupled with a conspicuous recovery of enzymatic activity.

This protection may once again represent a pro-survival mechanism which makes tumors/adapted cell more resistant to stress stimuli and therefore able to proliferate [53].

### Conclusions

In order to identify putative molecular marker(s) able to correlate with clinical and biological evolution of cervical dysplastic lesions, we analyzed the expression of stress response proteins and the pattern of oxidative adducts on DNA and proteins in a set of normal, dysplastic and neoplastic cervical tissues infected with HPV-16 mostly in an integrated status.

**Figure 6:**
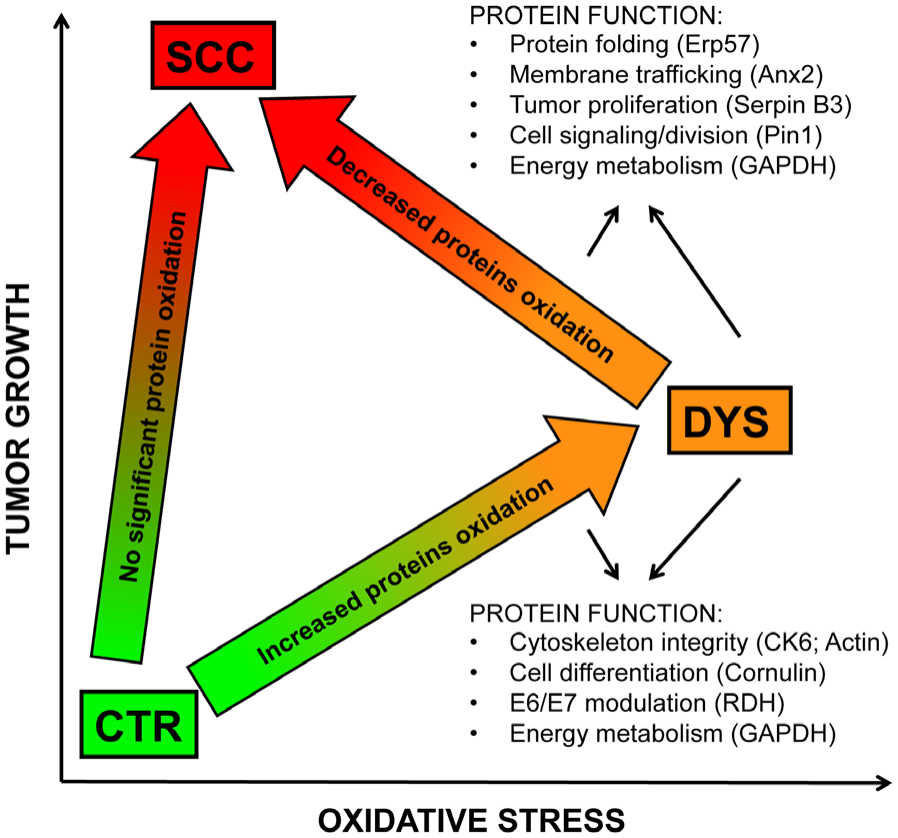
Proposed scenario of putative interplay between oxidative stress and tumor development. Function of the proteins showing altered oxidation in both DYS and SCC are listed. DYS is characterized by an increased oxidative environment, compared with both control and SCC tissues.

The up-regulation of stress protein markers indicated that an increased oxidative environment occur both in dysplastic and neoplastic tissues. However, in dysplastic tissues this condition resulted in oxidative modification of DNA and of proteins involved in cell morphogenesis and terminal differentiation such as CK6, actin, cornulin, RDH and GAPDH, providing the conditions for the neoplastic progression. Conversely cancer tissues seem to gain an improved control on oxidative damage as shown by the selective reduction of carbonyl adducts on key detoxifying/pro-survival proteins such as ERp57, Anx2, Serpin B3, Pin1 and GAPDH (figure 6).

Further studies are needed to better understand the effects of protein oxidation on cell transformation and cancer promotion. The comprehension of these phenomena may also concur to improve current clinical protocols for screening and for prognostic evaluation of cervical lesions.
